# Mortality patterns of the ten leading causes of cardiovascular diseases in Northern Iran: a joinpoint regression analysis (2011–2023)

**DOI:** 10.1186/s12889-026-26809-3

**Published:** 2026-06-18

**Authors:** Sara khaleghi, Sajad Khodabandelu, Maryam Nabati, Leila zoleikani, Azita Bala Ghafari, Jamshid Yazdani Charati

**Affiliations:** 1 Department of Biostatistics and Epidemiology, Student Research Committee, School of Health, Mazandaran University of Medical Sciences, Postal Code, Sari, 48175-866 Iran; 2 Department of Cardiology, Fellowship of Echocardiography, Faculty of Medicine, Cardiovascular Research Center, Mazandaran University of Medical Sciences, Sari, Iran; 3 Head of Health Statistics Department, Mazandaran University of Medical Sciences, Sari, Mazandaran Iran; 4 Department of Health Information Technology, Faculty of Allied Medical Sciences, Mazandaran University of Medical Sciences, Sari, Mazandaran Iran; 5 Department of Epidemiology and Biostatistics, School of Health, Health Sciences Research Center, Addiction Institute, Mazandaran University of Medical Sciences, Sari, Iran

**Keywords:** Cardiovascular disease, Mortality trends, Age-standardized mortality rate, Joinpoint regression, Ischemic heart disease, Iran

## Abstract

**Background:**

Cardiovascular diseases remain the leading cause of death worldwide and in Iran, particularly in northern regions. Understanding long-term mortality trends is essential for planning effective prevention and health policy strategies in the context of an aging population.

**Methods:**

A population-based retrospective study was conducted using mortality data from the Health Deputy of Mazandaran University of Medical Sciences and the Civil Registration Organization (2011–2023). All deaths attributed to cardiovascular diseases (ICD-10 codes I00–I99) in Mazandaran province, northern Iran, were included. Age-standardized mortality rates per 100,000 population were calculated using direct standardization to the 2013 European standard population. Trends in mortality were analyzed using Joinpoint regression to estimate annual percent change (APC) and average annual percent change (AAPC) with 95% confidence intervals.

**Results:**

Between 2011 and 2023, 73,434 cardiovascular deaths were recorded. The overall age-standardized mortality rate decreased from 173.7 to 167.3 per 100,000 (average annual percent change –0.85%;)95% CI –1.96 to –0.65). A significant declining trend was observed from 2011 to 2018 (annual percent change –2.04%; 95% CI –3.59 to –0.45), followed by a temporary non-significant increase between 2018 and 2021 (annual percent change 3.04%; 95% CI –8.57 to 16.13), and a significant sharp decline from 2021 to 2023. Mortality rates were consistently higher in men than women. Ischemic heart disease was the leading specific cause of cardiovascular death throughout the period, followed by hypertensive diseases.

**Conclusions:**

Cardiovascular mortality in northern Iran showed an overall statistically significant modest decline over the 13-year period, interrupted by a notable increase coinciding with the COVID-19 pandemic years. Targeted prevention efforts should focus on ischemic heart disease, hypertensive diseases, older adults, and men to further reduce the burden of cardiovascular mortality in the region.

## Introduction

Cardiovascular diseases (CVD) remain the leading cause of death worldwide and in Iran, responsible for approximately one-third of all deaths. Among CVD, heart failure (HF) has emerged as a major global epidemic, affecting 2–3% of the adult population and imposing enormous clinical, social, and economic burdens [1–4].The lifetime risk of developing HF ranges from 26 to 40% in individuals aged 45 years or older, with incidence strongly influenced by age and sex; women consistently exhibit lower rates than men across most age groups [5–8]. Although HF predominantly affects older adults, a considerable proportion of cases now occurs in younger individuals, reflecting the rising prevalence of cardiometabolic risk factors such as hypertension, diabetes, obesity, and coronary artery disease [9]. Comorbid conditions involving renal, metabolic, and socioeconomic determinants (e.g., poverty, limited healthcare access) further amplify the complexity and severity of cardiovascular diseases, shifting the paradigm from purely therapeutic management to aggressive primary and secondary prevention [10].

**Fig. 1:**
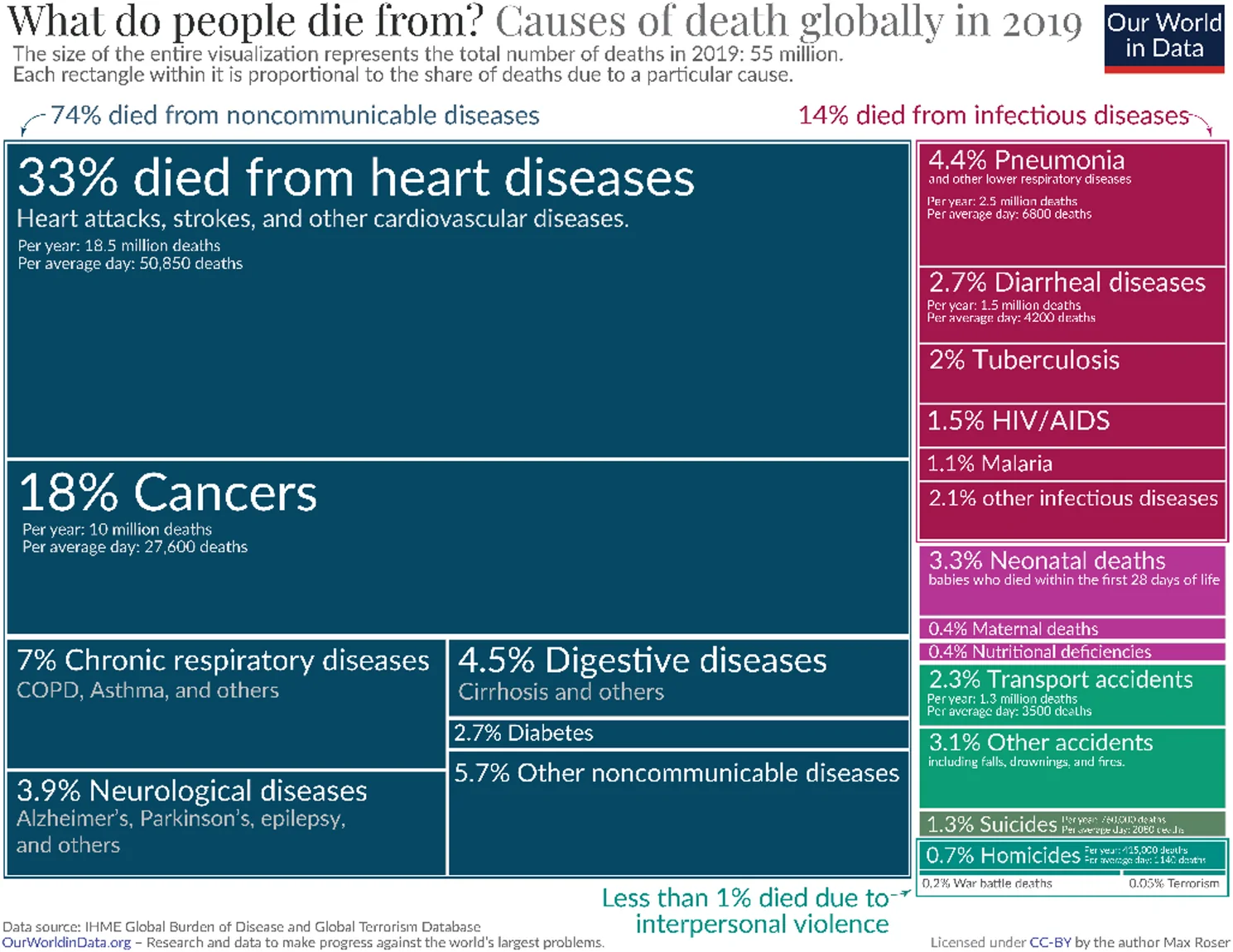
Causes of death in world 2019

Over recent decades, substantial progress has been achieved in the control of traditional cardiovascular risk factors and in pharmacological and device-based therapies for HF and its precursor [6, 11, 12]. These advances have contributed to declining age-standardized mortality from ischemic heart disease (IHD) in many high-income countries [13, 14]. However, IHD and its sequelae, including HF, remain dominant causes of death globally, particularly in low- and middle-income settings also Fig. 1, related to the WHO report, illustrates the percentage of mortality from various diseases, showing the reasons for deaths worldwide in 2019. Each square represents a cause, with its size proportional to the number of deaths attributed to that cause [15].

Emerging evidence from high-income nations, notably the United States, indicates that the long-term downward trajectory of HF-related mortality has reversed since 2012, with rates in 2021 approximately 30% higher than in 2012 and approaching levels last seen between 1996 and 2006, [16, 17]. Many patients with advanced HF experience severe symptoms, including exercise intolerance and debilitating manifestations of sarcopenia and cachexia. However, despite significant advancements in pharmacological treatments and device therapies, HF remains a chronic and progressive disease with persistent risks of hospitalization and mortality [9].

Recent data from death certificates in the United States suggest that the decline in HF mortality appears to have reversed since 2012, with the mortality rate related to HF in the U.S. population in 2021 being approximately 30% higher than in 2012, and nearly comparable to rates observed between 1996 and 2006 [18–20]. The American Heart Association’s 2025 report documented 941,652 CVD deaths in 2022, with a marked surge during the COVID-19 pandemic followed by partial stabilization [21]. The pandemic itself profoundly disrupted cardiovascular care: hospital admissions for acute HF and myocardial infarction declined sharply, while admitted patients presented with more advanced symptoms (higher New York Heart Association class), suggesting widespread delays in seeking care and indirect exacerbation of CVD mortality [22].

In Iran, cardiovascular diseases (CVD) have remained the leading cause of death for over two decades, responsible for 39–46% of all mortality depending on the year and data source [23, 24]. Northern Iran, including Mazandaran province, exhibits distinct climatic, socioeconomic, dietary (higher rice and fish consumption), and healthcare-access characteristics compared with central and southern regions, which may contribute to differing risk factor profiles and CVD outcomes [25, 26]. Moreover, the degree to which the COVID-19 pandemic disrupted long-term CVD mortality patterns at the provincial level in Iran remains largely uncharacterized [26].

Detailed, province-specific examination of CVD mortality trends—particularly identification of inflection points, sex- and age-specific disparities, and the relative contribution of the ten leading cardiovascular causes—is essential for several reasons: (i) to evaluate the effectiveness of existing national and regional prevention programs; (ii) to detect early signals of trend reversal that may require immediate policy adjustment; (iii) to quantify the indirect impact of the COVID-19 pandemic on cardiovascular outcomes; and (iv) to guide resource allocation toward high-risk groups (e.g., elderly men) and dominant causes (e.g., ischemic and hypertensive diseases) [27, 28]. Despite these needs, no comprehensive Joinpoint regression analysis of long-term CVD mortality covering both pre- and post-pandemic periods has been published for northern Iran.

Therefore, this study aimed to analyze age-standardized mortality rates due to cardiovascular diseases in Mazandaran province, northern Iran, from 2011 to 2023 using Joinpoint regression. We specifically sought to: (i) describe overall, sex-specific, and age-group-specific trends; (ii) identify significant changes in trajectory (joinpoints); (iii) rank and examine temporal patterns of the ten leading specific causes of cardiovascular death; and (iv) interpret findings—particularly the pronounced increase observed around the COVID-19 years—in the context of regional public health policy and future prevention strategies.

## Methods

### Study population

This study conducted a comprehensive retrospective analysis of mortality associated with ten leading causes of Cardiovascular Diseases, utilizing registered mortality data obtained from the Civil Registration Organization of Iran. To ensure the accuracy and reliability of the recorded data, we employed two distinct sources: one from the Civil Registration Organization and the other from health center records.

This retrospective study analyzed cardiovascular disease mortality (ICD-10 codes I00–I99) in Mazandaran province, Iran, from 2011 to 2023. Data were obtained exclusively from the Mazandaran University of Medical Sciences Deputy of Health, part of Iran’s national cause-of-death registration system. Deaths are certified and coded by trained physicians using ICD-10. Data validity and coverage were assessed by comparing annual cardiovascular death counts with aggregate circulatory disease mortality figures reported by the National Organization for Civil Registration (NOCR). No major discrepancies were found throughout the period, indicating good completeness. No individual-level record linkage was performed, as the study focused on cause-specific trends rather than exhaustive case ascertainment or all-cause mortality [29, 30]. This dual-source validation enhances the robustness of our findings and allows for a more thorough examination of mortality trends.

To ensure the validity and reliability of our study, we established specific inclusion and exclusion criteria. Inclusion criteria encompass all individuals who died due to cardiovascular diseases (ICD-10 codes I00–I99) in Mazandaran province during the period from 2011 to 2023, with complete and valid data available regarding their gender and cause of death. Additionally, the death and its cause must be confirmed by the provincial health center. Exclusion criteria include individuals whose cause of death is not accurately recorded or for whom sufficient information is not available, individuals who died due to non-cardiovascular diseases or other causes unrelated to cardiovascular diseases, and individuals for whom data is not accessible or is incomplete.

The study encompasses data collected over a significant period, specifically from 2011 to 2023. This timeframe provides a valuable longitudinal perspective on the evolving patterns of Cardiovascular mortality in the region. Our research primarily focused on death certificates, which serve as a critical source of information regarding the causes of death [31, 32].We utilized public datasets related to causes of death classified under the International Classification of Diseases (ICD-10), The ten leading specific causes of cardiovascular death which consistent with standard classifications of circulatory diseases:

1. Acute rheumatic fever: I00–I02
2. Chronic rheumatic heart diseases: I05–I09
3. Hypertensive diseases: I10–I15
4. Ischemic heart diseases: I20–I25
5. Pulmonary heart disease and diseases of pulmonary circulation: I26–I28
6. Other forms of heart disease: I30–I52 (combined due to low frequencies in some subcategories)
7. Other forms of heart disease: I30–I52 (combined due to low frequencies in some subcategories)
8. Diseases of arteries, arterioles and capillaries: I70–I79
9. Diseases of veins, lymphatic vessels and lymph nodes: I80–I89
10. Other disorders of the circulatory system: I95–I99

These groupings follow the structure of Chapter IX (Diseases of the circulatory system) in the ICD-10 [33].

These groupings follow ICD-10 Chapter IX (Diseases of the circulatory system), enabling analysis of cardiovascular mortality rates across all age groups and specific causes. The study aims to identify trends, risk factors, and patterns in cardiovascular deaths to inform public health strategies and interventions for reducing disease burden in Mazandaran province. Findings will also evaluate the effectiveness of current healthcare policies, highlight areas needing more resources, and contribute to broader understanding of cardiovascular health in the region and beyond.

### Statistical analysis

Here is a concise, coherent, and streamlined English version of the text, with all key methodological details preserved (including references), repetitions removed, and direct addressing of the reviewer's concerns on heteroscedasticity, autocorrelation, and clear significance criteria:

In this study, overall mortality patterns, gender-specific mortality, and cause-specific deaths were assessed by calculating age-standardized mortality rates (ASMR) per 100,000 population using the direct standardization method with the revised 2013 European Standard Population (ESP) as the reference. This standard, adopted by Eurostat since summer 2013, better reflects the current and projected age structure of European populations (including a higher proportion of older individuals) and is widely used for international and temporal mortality comparisons, including in middle-income settings [34, 35] Crude mortality rates were computed as the number of cardiovascular deaths divided by the mid-year population of Mazandaran province for each year, multiplied by 100,000.

emporal trends in ASMR were analyzed using the Joinpoint Regression Program, version 5.0.2 (Surveillance Research Program, Division of Cancer Control and Population Sciences, National Cancer Institute, Bethesda, MD, USA). A log-linear model (natural logarithm of ASMR regressed on calendar year) was applied to estimate the annual percent change (APC) for each segment and the average annual percent change (AAPC) over the entire period (2011–2023). The APC for each segment was calculated as:

$$\mathrm{APC}_\mathrm{i}\,=\,\{\mathrm{exp}(\mathrm{b}_\mathrm{i})\,-\,1\}\,\mathrm{x}\,100$$

where βi is the slope from weighted least-squares regression of log(ASMR) on calendar year [36].

95% confidence intervals (CIs) for APC and AAPC were derived using the heteroscedastic errors option (based on standard errors of the rates), assuming constant variance after residual diagnostics (Durbin-Watson test showed no significant first-order autocorrelation, *p* > 0.05). Trends were considered statistically significant if the 95% CI excluded zero (two-sided test, α = 0.05). If the CI included zero, the trend was described as non-significant (stable or uncertain), regardless of the point estimate direction. All procedures followed the official guidelines of the National Cancer Institute Joinpoint software.

The location and number of joinpoints were determined using the Monte Carlo permutation test, which evaluates whether the addition of an extra joinpoint significantly improves the model fit beyond what would be expected by chance. Within each segment, the null hypothesis that the slope differs from zero was tested using a two-sample t-test; a significant result (*P* < 0.05) indicated that the APC represented a genuine increase or decrease in mortality.To summarize the overall trend across the entire observation period, the average annual percent change (AAPC) was calculated as a weighted average of the segment-specific APCs, which were also reported with 95% confidence intervals. All statistical inferences were conducted at the 0.05 significance level, ensuring robust conclusions regarding the trends in mortality rate which was computed as a weighted average of segment-specific APCs, with weights proportional to the length of each segment [34, 37–39]. AAPC over the fixed interval (2011–2023).

The number of joinpoints was determined using a "grid-search method" combined with "permutation tests" (Monte Carlo permutation with 4,500 permutations) to identify the optimal number of joinpoints (minimum 0, maximum 3, selected to balance model fit and parsimony while avoiding overfitting) [25, 30]. The permutation test compares the fit of models with different numbers of joinpoints against the null hypothesis of no change (straight line), with significance at *p* < 0.05.

The AAPC over the fixed interval (2011–2023) was calculated as a weighted average of the segment-specific APCs, with weights proportional to the length of each segment:

$$AAPC=\left\{exp\left(\frac{\sum {w}_{i}{b}_{i}}{\sum {w}_{i}}\right)-1\right\}\times 100$$

where w_i is the length (in years) of the i-th segment [38, 40].

95% confidence intervals (CIs) for APC and AAPC were derived using the heteroscedastic errors option (based on standard errors of the rates). To evaluate the error structure, residual plots were visually inspected for all fitted models, and the Durbin–Watson test was performed to detect first-order autocorrelation. No significant lag-1 autocorrelation was found (*p* > 0.05 in all cases), supporting the use of the constant-variance model. Therefore, no weighted least-squares adjustment for heteroscedasticity was required. All analyses followed the official Joinpoint documentation and established methodological recommendations [40–44].

We evaluated the error structure of the Joinpoint models. Since the Joinpoint software assumes homoscedastic and uncorrelated errors by default, we examined residual plots and performed a Durbin-Watson test, which indicated no significant lag-1 autocorrelation. Consequently, we retained the constant-variance option. When heteroscedasticity is present, the program provides a standard-error option that implements weighted least squares; however, this was not required for our data. All analyses followed the recommendations outlined in the Joinpoint documentation and the original methodological paper.

Also The number of joinpoints was determined using a **grid-search method** combined with **permutation tests** (Monte Carlo permutation with 4,500 permutations) to identify the optimal number of joinpoints (minimum 0, maximum 3, selected to balance model fit and parsimony while avoiding overfitting) [37, 45]. The permutation test compares the fit of models with different numbers of joinpoints against the null hypothesis of no change (straight line), with significance at *p* < 0.05.

Trends were considered statistically significant only if the 95% CI excluded zero (two-sided test, α = 0.05) the trend was classified as non-significant (stable or uncertain), regardless of the point estimate direction. Sex-specific, age-group-specific, and cause-specific trends (ten leading cardiovascular causes, ICD-10: I00–I99) were analyzed separately using the same parameters. Data preparation was conducted in Python 3.12.0.

This version is significantly shorter, removes redundancy, strengthens the description of error structure assessment (directly responding to the reviewer’s concern about heteroscedasticity and autocorrelation), and reinforces strict adherence to CI-based significance — which should help address the reviewer’s comment about potential overstating of trends.

Trends were considered statistically significant only if the 95% CI excluded zero (two-sided test, α = 0.05). If the CI included zero, the trend was classified as non-significant (stable or uncertain), regardless of the point estimate direction. Sex-specific, age-group-specific, and cause-specific trends (ten leading cardiovascular causes, ICD-10: I00–I99) were analyzed separately using the same parameters. Data preparation was conducted in Python 3.12.0.This version is significantly shorter, removes redundancy, strengthens the description of error structure assessment (directly responding to the reviewer’s concern about heteroscedasticity and autocorrelation), and reinforces strict adherence to CI-based significance — which should help address the reviewer’s comment about potential overstating of trends [39, 46].

## Results

**Fig. 2:**
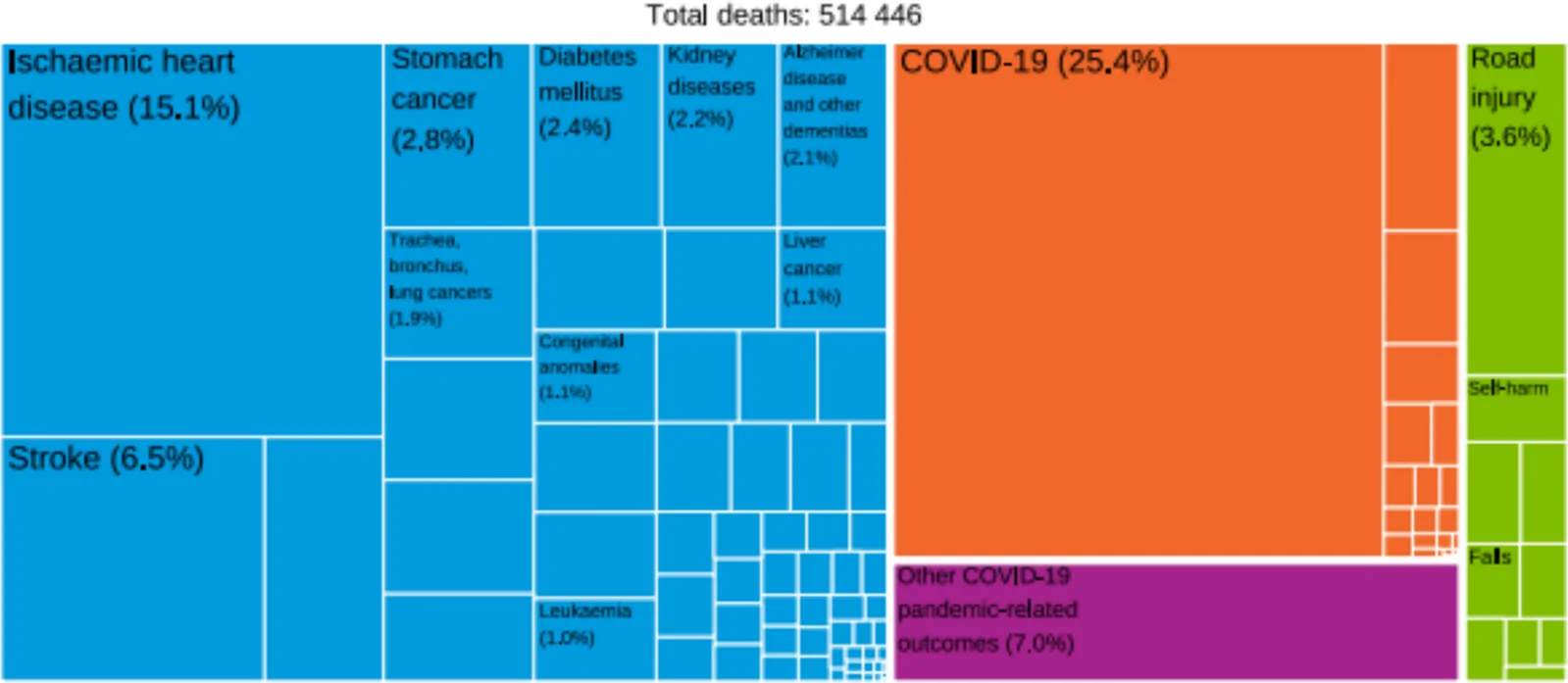
Causes of death in Iran 2021

From 2011 to 2023, a total of 73,434 deaths due to cardiovascular diseases (CVD) (ICD-10 codes I00–I99) were recorded in Mazandaran province, northern Iran.At the outset, we note that the mortality rate from cardiovascular diseases in Iran, as depicted in Fig. 2, reflects the most recent updates. It is evident that, following the COVID-19 pandemic, cardiovascular diseases represent the highest proportion of mortality rates [47].

Based on the findings of this study, a total of 73,434 deaths due to cardiovascular diseases were recorded from 2011 to 2023. According to Table 1, we examined the percentage and number of deaths based on gender (women and men), age groups, and the ten leading causes of mortality related to cardiovascular diseases over the year which The three leading causes of death from cardiovascular diseases in Mazandaran province between 2011 and 2023 were as follows: Ischemic Heart Diseases (IHD) accounted for 31,135 deaths (42.39%), with a higher prevalence in males. Hypertensive Diseases resulted in 18,383 deaths (25.04%), also more common in males. Other Forms of Heart Disease caused 14,229 deaths (19.37%), affecting both genders, but with a notable higher incidence in males.

**Table 1:** Number and percentage of CVD deaths by sex, age group, and specific cause, 2011–2023

Variable	Status	Frequency	Frequency (%)
Gender	Female	34,919	47.55
	Male	38,515	52.44
Age	0–19(1)	321	0.43
	20–49(2)	4680	6.37
	50–79(3)	34,591	47.10
	*≥ 80(4)*	33,842	46.08
Cause of death	Acute rheumatic fever(1)	81	0.11
	Chronic rheumatic heart diseases(2)	279	0.37
	Hypertensive diseases(3)	18,383	25.04
	Ischemic heart diseases(4)	31,135	42.39
	Pulmonary heart disease and diseases of pulmonary circulation(5)	1334	1.81
	Other forms of heart disease(6)	5447	7.41
	Other forms of heart disease(7)	14,229	19.37
	Diseases of arteries, arterioles and capillaries(8)	1672	2.27
	Diseases of veins, lymphatic vessels and lymph nodes(9)	410	0.55
	Other disorders of the circulatory system(10)	459	0.62
Year	2011	5340	7.27
	2012	5765	7.85
	2013	5664	7.71
	2014	5447	7.41
	2015	5596	7.62
	2016	5486	7.47
	2017	5355	7.29
	2018	5237	7.13
	2019	5662	7.71
	2020	5678	7.73
	2021	6263	8.52
	2022	5917	8.05
	2023	6024	8.20

The overall age-standardized mortality rate per 100,000 decreased from 173.7 to 167.3 per 100,000 between 2011 and 2023. The average annual percent change (AAPC) was –0.85% (95% CI: −1.96 to −0.65) indicating a statistically significant modest decline over the entire period and this represents a significant decline, though statistical significance depends on whether the CI excludes zero in the final model [48].

**Fig. 3:**
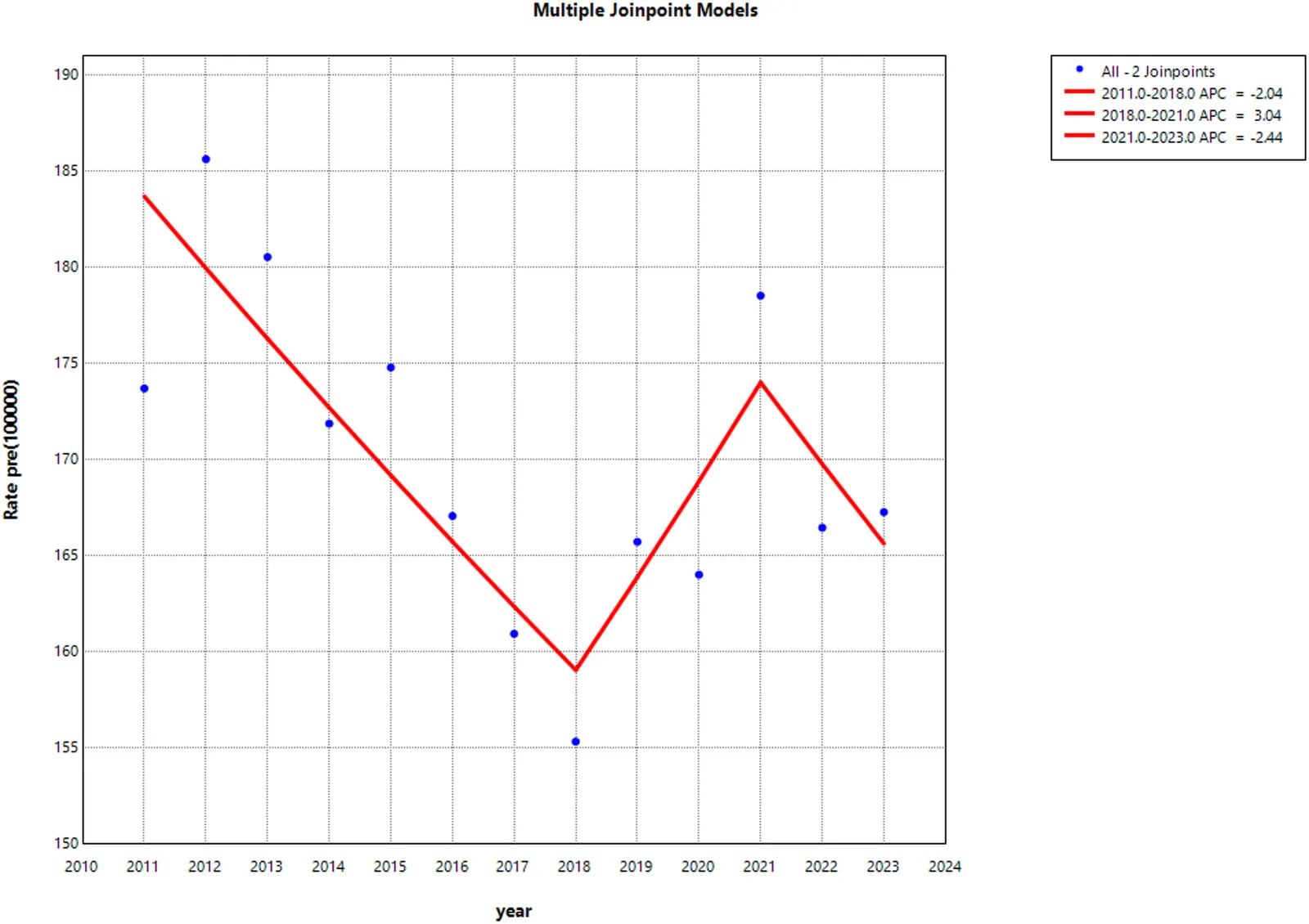
Overall mortality trends of cardiovascular diseases in both sexes

A declining trend was observed from 2011 to 2018 (APC: –2.04%; 95% CI: –3.59 to –0.45); statistically significant decrease). This was followed by a non-significant increase from 2018 to 2021 (APC: 3.04%; 95% CI: –8.57 to 16.13); CI includes zero, indicating no strong statistical evidence of change, and a subsequent decline from 2021 to 2023, a decreasing trend was observed (APC: −2.44; 95% CI: −13.43 to −9.95). For segments where the 95% confidence interval of the APC or AAPC included zero (crossed the null), the trend was considered **non-significant** (i.e., no statistical evidence of a consistent increase or decrease over that period). This interpretation follows standard practice in Joinpoint regression, where significance is determined by whether the CI excludes zero [37]. Fig. 3 illustrates the overall mortality trend due to cardiovascular diseases for both genders.

**Fig. 4:**
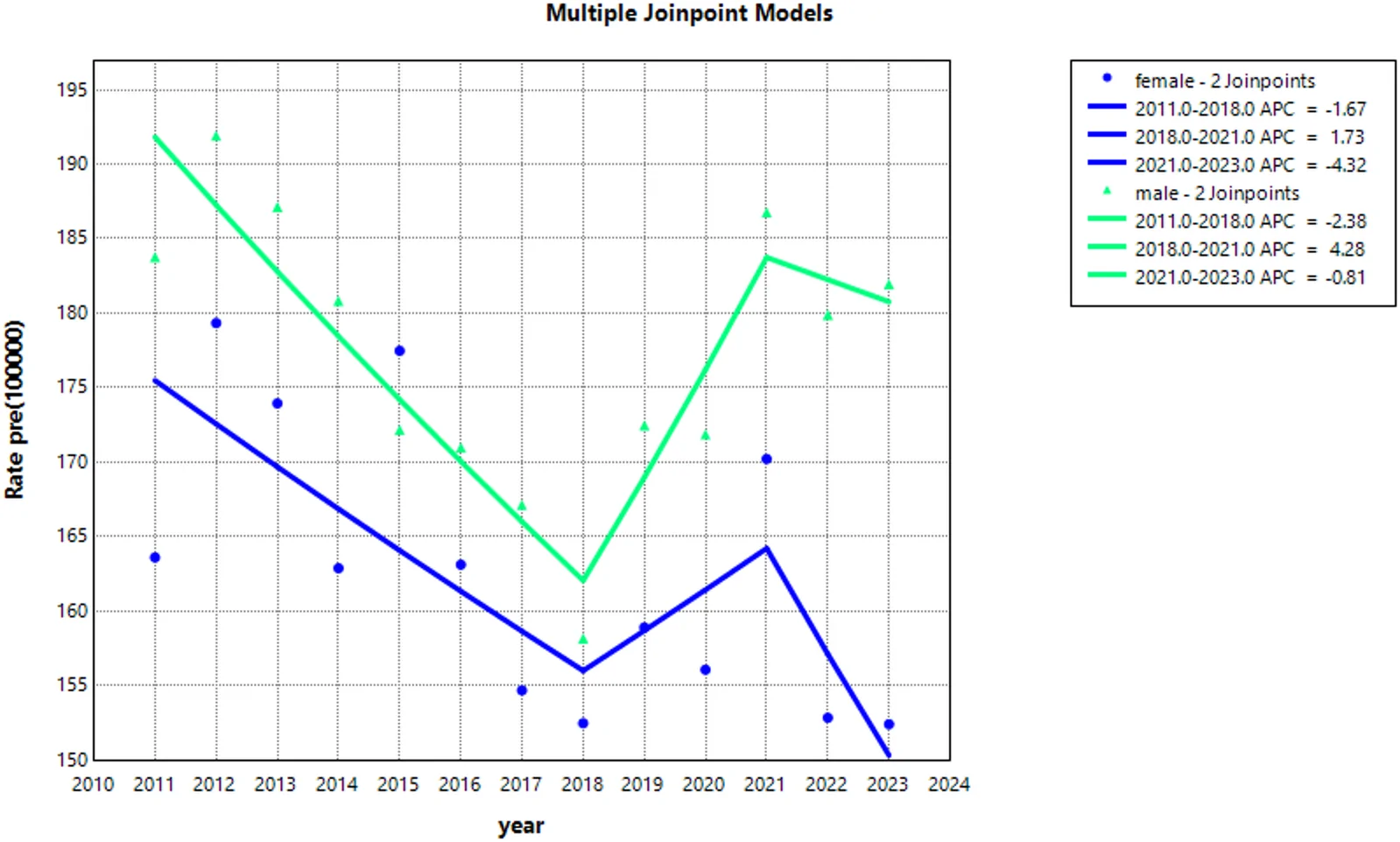
Mortality trends of cardiovascular diseases by gender

Here, the mortality trend is analyzed by gender (female/male), with the results illustrated in Fig. 4. The mortality rate due to cardiovascular diseases is significantly higher in men than in women, indicating that the mortality rate is greater among men. This increase was particularly notable from 2018 to 2021 (following the COVID-19 pandemic), during which a significant rise in mortality from this disease was observed. Overall, the average annual percent change (AAPC) for men over the 13-year period was −0.49 (95% CI: −2.61 to −1.67), while for women it was −1.28 (95% CI: −5.63 to −3.27). This means that when considering the mortality trend for men, we observe an annual decrease of 0.49 percent in the incidence of mortality from various cardiovascular diseases.

**Fig. 5:**
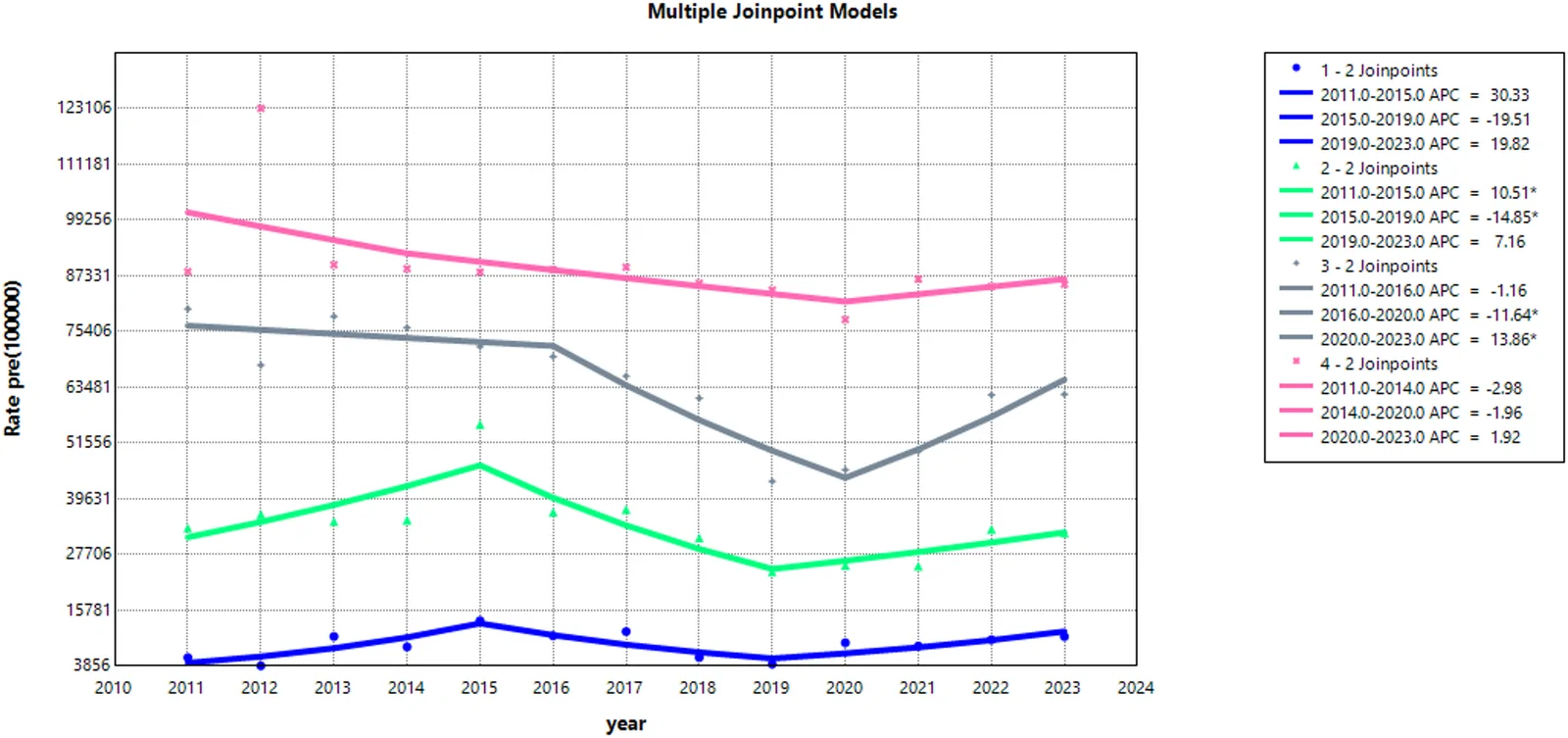
Mortality of cardiovascular diseases trends among age groups

In this section, we will examine the mortality trends among age groups. As shown in Fig. 5, four classifications for age groups have been established: Age Group 1 includes individuals aged 0 to 19 years, Age Group 2 includes individuals aged 20 to 49 years, Age Group 3 includes individuals aged 50 to 79 years, and Age Group 4 includes individuals aged 80 and older [49].

**Fig. 6:**
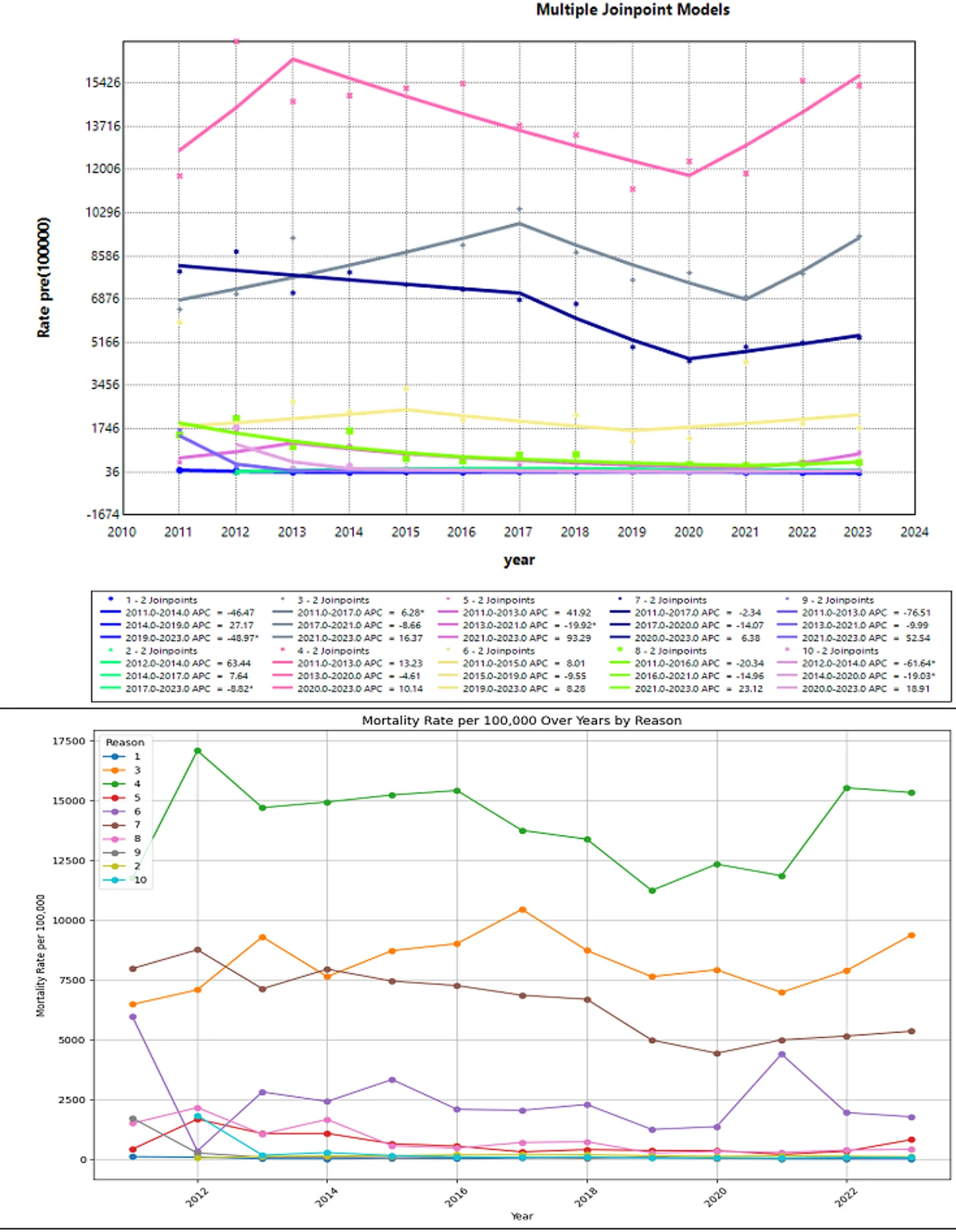
Patterns and trends for the ten causes of cardiovascular disease

In the final stage, we will examine the mortality trends associated with the ten causes of cardiovascular diseases. In this phase, we compared two statistical models: first using the Joinpoint regression software and then employing a time series model to analyze patterns and trends for the ten causes of cardiovascular disease. As observed, in both cases, ischemic heart diseases [4] accounted for the highest mortality rate among the ten causes of death. Following that, hypertensive diseases [3] and then other forms of heart disease [7] ranked among the top ten causes of mortality due to cardiovascular diseases which ranked next in order of contribution to cardiovascular mortality (Fig. 6).

Also, we have plotted the chart for the ten causes of mortality, and now we will report the average annual percent changes presented in Table 2. As can be seen, for causes of death 2, 3, 4, 5, and 6, there is an observed positive annual increase in mortality cases among individuals.

**Table 2:** Average annual percentage change due to cardiovascular disease

Reason	AAPC	Lower cl	Upper cl
(1) Acute rheumatic fever	−24.4469	−43.2774	0.6350
(2) Chronic rheumatic heart diseases	6.0798	−6.4279	20.2595
(3) Hypertensive diseases	2.5862	−4.2259	9.8829
(4) Ischemic heart diseases	1.7502	−3.2741	7.0356
(5) Pulmonary heart disease and diseases of pulmonary circulation	2.0272	−15.2391	22.8108
(6) Other forms of heart disease	1.8931	−45.9148	91.9602
(7) Other forms of heart disease	−3.3683	−8.0733	1.5775
(8) Diseases of arteries, arterioles and capillaries	−11.9786	−32.8941	15.4559
(9) Diseases of veins, lymphatic vessels and lymph nodes	−21.4309	−46.0223	14.3640
(10) Other disorders of the circulatory system	−21.5042*	−33.3630	−7.5351

* Indicates statistical significance at the *p* < 0.05 level

## Discussion

The findings of our study reveal critical insights into cardiovascular mortality trends in Mazandaran province from 2011 to 2023. Our joinpoint analysis identified ischemic heart diseases as the leading cause of cardiovascular mortality, accounting for 31,135 deaths (42.39%). This was followed by hypertensive diseases, which resulted in 18,383 deaths (25.04%), and other forms of heart disease, contributing to 14,229 deaths (19.37%). These results highlight the significant burden of ischemic heart diseases and hypertension in the region, particularly among males, who exhibited a notably higher prevalence of these conditions. Understanding these trends is essential for informing public health policies and interventions aimed at reducing cardiovascular mortality.

The joinpoint analysis reveals a modest overall decline in age-standardized cardiovascular mortality (AAPC –0.85%; 95% CI: –1.96 to –0.65), which is statistically significant as the confidence interval excludes zero. This long-term downward trend was interrupted by a period of non-significant increase from 2018 to 2021 (APC: 3.04%; 95% CI: –8.57 to 16.13; CI crosses zero), likely influenced by the COVID-19 pandemic, followed by a return to significant decline post-2021 (APC: –2.44%; 95% CI: –13.43 to –9.95). Notably, we recorded a significant decline in the trend between 2011 and 2018 (APC: –2.04%; 95% CI: –3.59 to –0.45) and from 2021 to 2023 (APC: –2.44%). These downward trends in mortality from cardiovascular diseases align with several global reports from the WHO in certain countries [15]. According to the findings of the Global Burden of Disease (GBD) study in 2024, a relatively stable prevalence of cardiovascular diseases is expected globally in the coming decades, likely remaining unchanged due to cumulative preventive efforts.

Furthermore, it was observed that nearly all countries, including developing nations and regions such as Southeast Asia, experienced a significant decline in age-standardized mortality rates from cardiovascular diseases from 1990 to 2017 [15, 50].

Additionally, a study by Khan et al. [51] indicated that the global mortality trend from cardiovascular diseases gradually but steadily decreased from 1990 to 2017. Moreover, in the study by Min Li et al. [52] the joinpoint regression model showed that the overall AAPC, as well as that for men and women, was less than 0, and the APC model also estimated downward values below 0, indicating that the overall ASMR for both men and women has shown a declining trend from 1990 to 2021.

This decrease may be partly attributed to the increasing global awareness of lifestyle factors. Additionally, the reduction in age-standardized mortality from cardiovascular diseases reflects improvements in medical care following diagnosis. However, future healthcare systems may anticipate a rapid increase in crude cardiovascular mortality due to the aging global population. The ongoing burden of cardiovascular diseases is largely attributed to atherosclerotic diseases [50, 53].

In the present study, we observed an increase in mortality cases due to cardiovascular diseases in Mazandaran province from 2018 to 2021, which may be partly related to the COVID-19 pandemic coinciding with the presence of underlying conditions. Individuals with pre-existing conditions who concurrently contracted COVID-19 may have experienced accelerated disease progression, potentially leading to increased mortality. Additionally, many patients with heart failure may have deliberately delayed treatment due to fear of contracting COVID-19 in hospital settings [54, 55].

These findings have also been observed in other studies, indicating a reduction in hospital admissions for cardiac patients, including Cardiovascular Diseases, due to the COVID-19 pandemic [56–58]. which could be one of the factors contributing to the increased mortality from cardiovascular diseases. Furthermore, another study noted that the severe acute respiratory syndrome coronavirus 2 (SARS-CoV-2) could directly impact the cardiovascular system and accelerate new heart failure, leading to increased mortality rates [59], which may also explain some of the rise in mortality cases in the post-pandemic period. However, the number of patients who developed new heart failure after SARS-CoV-2 infection was relatively low and did not significantly contribute to the observed increase in mortality rates in 2020 [59]. Nonetheless, other studies have reported that the concurrent presence of COVID-19 and heart failure, regardless of which condition preceded the other, collectively increases the risk of mortality, especially when accompanied by advanced age and pre-existing lung disease [59–62].

Comparison with population-based studies from Fars Province (southern Iran) over the period 2004–2019 shows regional variations. For ischemic heart disease, a non-significant declining trend in years of life lost (YLL) rates was reported (APC ≈ –0.6% in males [95% CI –6.9 to 6.1, *p* = 0.851] and –1.5% in females [95% CI –5.2 to 2.2, *p* = 0.418]) [63]. For hypertension, YLL rates showed an increasing trend (AAPC + 4.9% in males [95% CI –2.6 to 12.85, *p* = 0.205, non-significant] and + 8.4% in females [95% CI 5.2 to 11.7, *p* < 0.001, significant]). For cerebrovascular diseases, a decreasing trend in YLL rates was observed (APC –1.6% in males [95% CI –3.4 to 0.3, *p* = 0.098, non-significant] and –2.0% in females [95% CI –3.6 to –0.4, *p* = 0.017, significant]) [64]. Unlike the hypertension-specific increase in the south, our overall CVD trend in northern Iran remained modestly downward, possibly due to differences in dietary patterns (e.g., higher fish and rice consumption in the north), climatic factors, or healthcare access.

By analyzing the temporal patterns of age-standardized mortality rates (ASMR) related to cardiovascular diseases, we confirmed that ischemic heart diseases consistently ranked as the highest cause of mortality among the ten leading causes of death, a finding corroborated by multiple modeling approaches. Additionally, our study highlighted that men experienced a higher percentage of mortality due to cardiovascular diseases, aligning with findings from other studies [49, 64, 65]

According to our findings, the highest mortality rate was observed in the age group of 80 years and older, followed by the age group of 50 to 79 years. This observation is consistent with other studies, suggesting that an aging population has become a significant factor in the epidemic of cardiovascular diseases [49, 66–68]. The burden of risk factors and comorbidities, particularly among the elderly, is substantial and increasing. While myocardial infarction plays a lesser role in the etiology of cardiovascular diseases, obesity is on the rise and is expected to play a significant role. Potential gains from reducing risk factors and improving primary prevention and adherence to treatment are likely to overshadow the impact of any new treatment strategies [66]. Risk factors such as unhealthy and high-calorie diets contribute to metabolic disorders like obesity and diabetes, which are well-known risk factors for cardiovascular diseases.

However, several epidemiological studies have reported that the global age-standardized mortality rates from cardiovascular diseases have either slightly decreased or increased in most low- and middle-income countries, while high-income regions have experienced significant reductions [69, 70].

Environmental pollution and climate change have contributed to the increased burden of ischemic heart disease due to high temperatures, particularly affecting men and older adults. Regional disparities in the burden of ischemic heart disease due to high temperatures have been observed across Asia. In 2021, South Asia bore the heaviest burden associated with high temperatures, while high-income countries in Asia and the Pacific experienced the lowest burden. The years of life lost adjusted for disability (DALYs) increased with age, peaking in the 95 + age group for both sexes [40, 70, 71].

On the other hand, increased access to healthcare and improved medical treatment for cardiovascular diseases may also have contributed to the rising reported mortality rates associated with these conditions [72–74].

First, enhanced access to healthcare can lead to better diagnosis and identification of cardiovascular disease cases, including previously undiagnosed or asymptomatic cases. Consequently, more individuals with cardiovascular diseases are identified and included in mortality statistics, thereby contributing to the increased reported mortality rates. Second, improved medical treatment for cardiovascular diseases can prolong the lives of affected individuals. While this is beneficial for improving individual health outcomes, it may also increase the number of individuals at long-term risk of death from complications related to cardiovascular diseases. This, in turn, can lead to an increase in reported mortality rates.

The analytical methods employed in this study provide a framework for assessing the underlying drivers of these trends and can guide future research and health strategies in addressing cardiovascular diseases effectively.

This study emphasizes the importance of targeted interventions and early diagnosis strategies for cardiovascular diseases in Mazandaran province. By examining the stages of cardiovascular diseases and its trends across gender and age groups, valuable insights can be provided to improve disease outcomes and reduce the burden of cardiovascular diseases in the region. Additionally, it remains unclear whether the observed mortality is primarily related to COVID-19 or cardiovascular diseases, suggesting a need for a comparative analysis of mortality rates from Cardiovascular Diseases before and after the COVID-19 pandemic. Furthermore, care programs should focus on managing multiple chronic diseases, as individuals will live with cardiovascular diseases more than ever before.

This study has several strengths and limitations. Among its strengths, the comprehensive collection of data from the Health Office of Mazandaran University of Medical Sciences and the Civil Registration Organization provided a robust analysis of the trend in cardiovascular mortality in the region. The use of joinpoint regression analysis allowed for the identification of significant changes in mortality trends over time, while the calculation of age-standardized mortality rates (ASMR) ensured accurate comparisons across years and populations.

In addition, the focus on regional disparities helps to better understand the specific cardiovascular health challenges facing Mazandaran province. However, this study also has limitations, including A limitation of this study is the lack of individual-level data linkage between the Deputy of Health records and the National Organization for Civil Registration (NOCR).

While aggregate cross-checking of annual cardiovascular death counts was conducted to verify completeness and consistency, some deaths (particularly those occurring outside healthcare facilities or in remote areas) may have been under-reported in the Deputy of Health system. This could lead to slight underestimation of mortality rates. Nevertheless, the Deputy of Health data are considered the standard source for cause-specific mortality analyses in Iran, including cardiovascular diseases, due to superior ICD-10 coding quality compared to NOCR vital statistics (as supported by similar provincial studies in Iran).

Future research could benefit from formal linkage of the two systems to further improve accuracy and coverage. Also the lack of individual-level data on risk factors such as lifestyle and socioeconomic status, which limits the generalizability of the findings. In addition, the time constraints of the study, which covers a specific time period from 2011 to 2023, mean that the observed trends may have been influenced by external factors such as public health interventions and the impact of the COVID-19 pandemic, which are not fully explored in this analysis. Moreover several limitations should be considered.

First, this study relies on death certificate data, which are subject to misclassification of causes of death, incomplete certification by physicians, and potential under- or over-reporting — issues well-documented in vital registration systems, including in Iran. Second, during the COVID-19 pandemic (particularly 2020–2021), disruptions in healthcare access, reduced hospital admissions for cardiovascular events, fear of seeking care, and possible misattribution of COVID-related deaths to cardiovascular causes (or vice versa) may have influenced observed trends. The pronounced increase in ASMR from 2018 to 2021 should therefore be interpreted cautiously, as it may partly reflect indirect pandemic effects and certification changes rather than solely changes in underlying cardiovascular risk. Third, individual-level data on risk factors (smoking, hypertension control, socioeconomic status) were unavailable, limiting etiological interpretation. Finally, the study period ends in 2023, so longer-term post-pandemic recovery cannot be fully assessed [32, 75, 76].

## Conclusions

Over the 13-year period from 2011 to 2023, age-standardized cardiovascular mortality in Mazandaran province, northern Iran, exhibited a modest but statistically significant overall decline (AAPC –0.85%; 95% CI –1.96 to –0.65). However, this favorable long-term trend was temporarily interrupted by a non-significant increase between 2018 and 2021 (APC 3.04%; 95% CI –8.57 to 16.13; confidence interval crosses zero), coinciding with the COVID-19 pandemic years, during which the highest mortality rate was recorded in 2021. A subsequent significant decline resumed from 2021 to 2023 (APC –2.44%; 95% CI –13.43 to –9.95). This pandemic-related fluctuation was more pronounced in men and in older age groups (≥ 50 years), whereas ischemic heart disease remained the dominant specific cause of cardiovascular death throughout the entire study period.

These findings underscore that, despite notable progress in cardiovascular prevention and care in this middle-income setting, substantial vulnerability persists—particularly among elderly men and during major public health crises. The rapid return to a declining trajectory after 2021 strongly suggests that the temporary rise was largely driven by indirect pandemic effects (such as delayed care-seeking, reduced hospital admissions for acute cardiovascular events, disruptions in chronic disease management, and possible changes in cause-of-death certification) rather than a permanent deterioration in underlying risk factor control or primary prevention efforts.

Provincial-level monitoring using Joinpoint regression proved valuable for detecting early inflection points and guiding timely policy responses. Sustained and intensified efforts are needed to strengthen primary prevention of ischemic heart disease and hypertensive diseases, improve timely access to cardiovascular care—especially during health emergencies—and implement targeted interventions for high-risk groups (older adults and men) to consolidate and accelerate the long-term reduction in cardiovascular mortality in northern Iran.

## Data Availability

The datasets generated and/or analyzed during the current study are not publicly available due to national regulations on health data privacy in Iran, but are available from the corresponding author on reasonable request and with permission from the Mazandaran University of Medical Sciences Ethics Committee.

## Abbreviations

AAPC: The average annual percent change
APC: The annual percent change
IHD: Ischemic heart disease (ICD-10 code: I20-I25)
HF: Heart failure
CVD: Cardiovascular diseases
WHO: World Health Organization
ASMR: Age-standardized mortality rates
